# Annual Papanicolaou screening for 5 years among human papillomavirus-negative women

**DOI:** 10.1186/1471-2407-13-379

**Published:** 2013-08-09

**Authors:** Karl Ulrich Petry, Franziska Rinnau, Gerd Böhmer, Bettina Hollwitz, Alexander Luyten, Nina Buttmann, Martin Brünger, Thomas Iftner

**Affiliations:** 1 Zentrum für Frauenheilkunde, Medizinische Hochschule Hannover (MHH), Carl-Neuberg-Street 1, 30625 Hannover, Germany; 2 Labor Wagner-Stibbe, Hannoversche Street 24, 31848 Bad Muender, Germany; 3 Klinik für Frauenheilkunde, Geburtshilfe und Gynäkologische Onkologie Klinikum Wolfsburg, Sauerbruchstr. 7, 38440 Wolfsburg, Germany; 4 Tumorepidemiologie, Universitäts Krebs Centrum Dresden am Universitätsklinikum Carl Gustav Carus an der Technischen Universität Dresden, Fetscherstraße 74, 01307 Dresden, Germany; 5 Center for Health and Human Sciences, Charité – Universitätsmedizin Berlin, Charitéplatz 1, 10117 Berlin, Germany; 6 Sektion für Experimentelle Virologie, Universitaetsklinikum Tuebingen, Elfriede-Aulhorn-Street 6, 72076 Tuebingen, Germany

**Keywords:** Annual papanicolaou smear, Cervical cancer screening, Human papillomavirus (HPV), HR-HPV DNA test, Screening intervals

## Abstract

**Background:**

Primary human papilloma virus (HPV) screening is more effective than cytology in reducing the risk of cervical cancer, but screening intervals should be extended in HPV-negative women. However, some Markov models predicted that long intervals are associated with an excess risk of cervical cancer. The aim of this analysis was to estimate the real-life risks and benefits of annual Papanicolaou (Pap) screening in HPV-negative women with normal cytology.

**Methods:**

Women with negative Hybrid Capture 2 (HC2) results and normal cytology at the time of inclusion in the Hannover HPV screening trial underwent annual Pap smears for 5 years. A subgroup was randomly selected for retesting with cytology, HC2, and colposcopy 60–68 months after recruitment.

**Results:**

Of 4236 women included, 3406 had at least one Pap smear, but only 1185 attended all five annual screening visits. The proportion of women with at least one abnormal smear was 14.4% in 60 months. The probability of abnormal smears increased continuously over time. No case of ≥ CIN2+ was observed during 5 years. Of 605 women selected for subgroup analysis, 292 agreed to be retested (48.3%). The rate of high-risk HPV at 60–68 months was 3.0% (9/296).

**Conclusions:**

The long-term risk of high-grade neoplasia after an initial negative HC2 test and normal cytology result was low, while the rate of false-positive abnormal Pap smears was significant and increased constantly over time. Pap smear screening of HPV-negative women more frequently than every 5 years could be potentially harmful and seems to be of little clinical value.

## Background

There is a high level of evidence that human papilloma virus (HPV) testing is more effective than cytology in reducing the risk of CIN3 and cervical cancer and allows for the extension of screening intervals up to 7 years [1,2]. Organized screening programs in Italy, Sweden, the Netherlands, and other countries are being transitioned from Papanicolaou (Pap) smear-based tests to primary HPV screening. The American Cancer Society recommends screening intervals of 5 years for women with negative high-risk HPV (HR-HPV) results and normal cytology while for cytology-based screening European guidelines discourage intervals of less than 3 years [3]. However concerns have been raised that longer intervals between screening rounds may compromise cervical cancer prevention. A Markov model, using data from the USA, estimated that increasing Pap smear screening intervals from 1 to 3 years after the last negative test in women aged 30–64 years would be associated with an average excess risk of cervical cancer of approximately 3 in 100,000 [4]. Similarly, a Markov Model for cervical cancer screening in Germany estimated that using intervals of more than 2 years for HPV screening would miss CIN3 lesions and may result in an excess risk of cervical cancer [5].

While there is general agreement among experts that the benefits of short-interval Pap smear testing may be outweighed by the risks of overdiagnosis, overtreatment and other kinds of harm, there is limited direct evidence to support this approach. In a prospective cohort study including 8466 women in Hannover and Tuebingen, Germany, we demonstrated that a negative HPV test result, even in combination with a positive Pap result, virtually excluded any risk of underlying high-grade disease [6]. The 5-year follow-up of this study was included in the joint European cohort study on the long-term predictive value of HPV testing and cytology to detect CIN3 or cancer [7]. However the European multicenter study did not analyze the frequency of abnormal screening results during follow-up or the possible correlation with the length of screening intervals. Within the European multicenter study, the Hannover cohort was the only sub-study with annual Pap smear follow-up for HPV-negative women with normal cytology at recruitment. Therefore, we have re-analyzed ‘real-life’ data from the Hannover cohort study to estimate the risks and benefits of annual Pap screening over 5 years in HPV-negative women with normal cytology.

## Methods

### Study population

Between December 1998 and September 2001, women (age 30 years or older) attending routine cervical cancer screening were recruited from 15 urban, suburban or rural, office-based gynecology practices in Hannover and the surrounding areas. Women were eligible for inclusion in the original trial [6] if they were attending for routine annual cervical cancer screening, were 30 years of age or older, had not undergone a hysterectomy, had no history of atypical cytology, CIN, or treatment for cervical disease in the preceding year, and were not currently pregnant. There was no upper age limit, but 94.6% of participants were aged 30–60 years old at recruitment. Written informed consent was obtained from the patients by the participating gynecologists. The study was approved by the local ethics committee at the University of Hannover. Here we report on the follow-up findings of women who had negative Hybrid Capture 2 (HC2) results and normal Pap smear findings at recruitment.

### Screening examinations and HPV testing

At the first gynecological examination, the cervix was visualized and a sample was taken for routine cervical cytology following the procedures normally used in each gynecological practice. A second sample was then obtained with a Digene Cervical Sampler (Medscan, Uppsala, Sweden), and suspended in 1 mL of specimen transport medium (STM/Digene Inc. Gaithersburg, MD, USA) for HPV DNA testing. Samples for HPV testing were stored at 4°C for a maximum of 4 weeks prior to testing at the University of Hannover. HPV testing was performed for high-risk types only, using the HC2 test (HR-HC2/Qiagen Inc.) following the manufacturer’s instructions. Samples were considered positive if they attained or exceeded the FDA approved threshold of 1.0 pg HPV DNA/mL.

### Cytological diagnoses

All cervical smears were analyzed at one of eight cytology laboratories routinely used by each participating gynecology office. In this study, smears were considered: a) ‘positive’ if any degree of cytological abnormality was observed (≥ Pap2w, which is equivalent to atypical squamous cells of undetermined significance [ASC-US] in the Bethesda system); b) ‘low-grade squamous intraepithelial lesion (LSIL) or more’ if classified as Pap3d (CIN1 or 2) or more. Pap3d (CIN 1) is equivalent to LSIL, whereas Pap3d (CIN2) is equivalent to high-grade squamous intraepithelial lesion (HSIL) in the Bethesda classification.

The ‘Pap2w’ (W = wiederholen or repeat) is a widely used category although it is not part of official German (Munich II) cytology classification [6]. Pap2w is equivalent to ASC-US, and according to national guidelines needs to be followed by repeat smears or HPV triage [8]. The cytology laboratories had not been informed about the study and therefore read the smears under routine screening conditions.

### Follow-up protocol

Women with negative HPV tests and normal Pap smear results (double negative) at recruitment were followed with annual Pap smear screening in private gynecology offices according to German guidelines. Pap smear results and all interventions related to cervical disease were reported for inclusion in the central study database.

After approximately 5 years’ follow up (50–68 months) a representative subgroup of women with initially double-negative test results (every seventh participant) was invited for colposcopy, Pap smears and HC2 retesting. Punch biopsies or endocervical curettage were performed if appropriate.

The aim of the follow-up subgroup analysis was to determine the proportion of women developing abnormal cytology and the proportion with HR-HPV infection at the end of the study.

### Statistics

The rationale for the selection of every seventh double-negative participant was based on our previous experience showing that in the first year of a study only 50% of such subjects will accept the invitation for repeat screening. In Germany in women aged 30+ years the average rate of CIN3+ is 0.3–0.9% and of CIN2+ 1.0–1.6% [9], therefore, we calculated that a sample of 300 women was required to estimate risk with sufficient precision in women with double-negative test results.

For data analysis we used SPSS Statistics version 20 (IBM Corporation, Armonk, NY, USA). The Kaplan-Meier method was used for survival analysis to determine the proportions of events of ≥ Pap2w during the follow-up period [10]. To investigate a potential influence of womens’ age, we applied a Cox regression model with age as a nominal covariate with four age categories of equal size [11].

## Results

### Findings at enrolment and during follow-up

**Figure 1:**
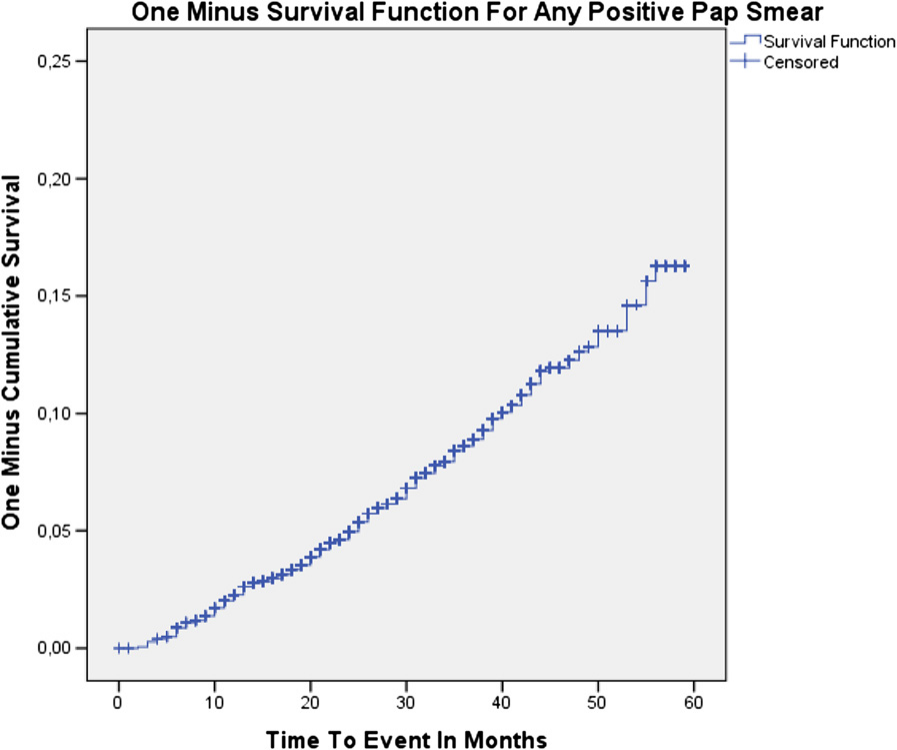
**Kaplan**-**Meier cumulative 1**-**minus survival curves showing the proportion of patients with abnormal cytology over time.**

At enrolment, 4236 of 4737 women had negative HC2 results and normal Pap smear findings, of whom 3406 (80.4%) had at least one further routine Pap smear in the following 1–6 years. Mean age was 40.8 years (SD 8.0). In total, 281 women developed an abnormal cytology, of whom 202 (mean age 40.3 years, SD 7.3) were diagnosed with ASC-US and 79 (mean age 42.2 years, SD 7.8) with LSIL or more during follow-up. Kaplan-Meier cumulative 1-minus survival curves (Figure 1) showed a continuous increase over time for any positive smear as well as for LSIL or more. During the 5-year follow-up, 1185 (34.8%) double-negative participants attended four or more annual Pap smear screening rounds in accordance with the German screening program. The proportion of women with abnormal Pap smear findings ranged from 2.2–3.5% per screening round. The cumulative proportion of women with at least one atypical Pap smear was 14.4% among those attending all five annual screening visits following enrolment. There was no significant association between women’s age and the cumulative proportion developing a LSIL or worse lesion (Table 1).

**Table 1:** **Association between age** (**in categories**) **and proportion developing a positive smear (≥ ASC**-**US)**

**Age group (years)**	**N**	**Odds ratio**	**95% Confidence intervals**
≤ 34	906		Reference
35–39	802	1.28	0.92–1.79
40–46	895	1.26	0.91–1.74
≥ 47	803	1.04	0.73–1.47

When consulted, we discouraged excisional treatment for all women with ASC-US and for most LSIL cases because of the assumed low risk of high-grade cervical disease. However, 15 patients were transferred for colposcopy during follow-up, 15 underwent diagnostic cold-knife conizations and three (aged 42, 51, and 54 years old at the time of the operation) had hysterectomies without colposcopy outside the study protocol because of persisting abnormal smears. No case of CIN2+ was reported among these 33 women with histological assessment.

### Proportion of abnormal Pap smears, HPV, and CIN at months 60–68

**Figure 2:**
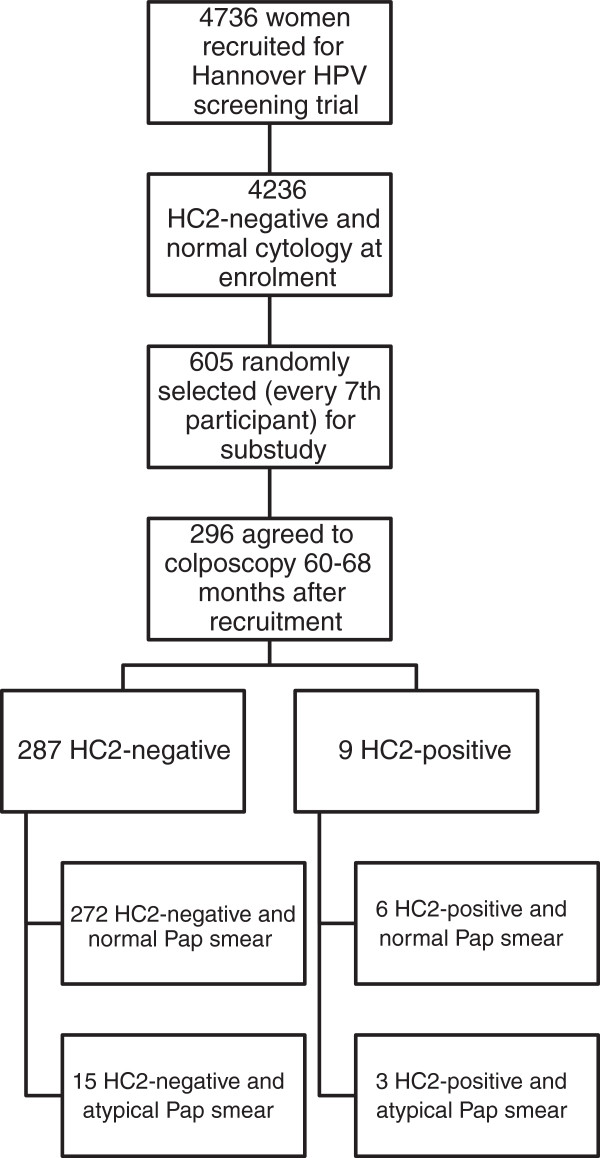
**Patient disposition and incidence of atypical Pap smear and positive HC2-tests among 296 women, 60**–**68 months after recruitment.**

From 605 women randomly selected from the population that tested HC2 negative and showed normal Pap smears at study entry, 296 (48.9%) agreed to undergo colposcopy 60–68 months after recruitment (Figure 2). Of these, 272 (91.9%) women retested negative for HR-HPV DNA using HC2 and had normal Pap smear results. In total, 18 women (6.1%) had Pap smears classified as ASC-US or LSIL, of whom 15 (5.1%) tested negative for HPV, while three (1.0%) had abnormal cytology and positive HC2 results. Overall, nine (3.0%) women had positive HC2 tests. Out of the total cohort of 296 women, 74 (25%) underwent punch biopsies at colposcopy, but no case of CIN 2+ was detected.

## Discussion

We re-analyzed data from a prospective cohort study to assess the benefits and harms of short-interval Pap screening in HPV-negative women. We did not find a single case of CIN2+ among 296 women who underwent careful assessment with colposcopy and, if necessary, biopsies at the end of the study, 60–68 months after recruitment. Furthermore, no case of CIN2 or more was found among the total study population (n = 3406) during Pap smear follow-up, including 33 women with histological assessment. We conclude that HPV screening allows early detection of clinically relevant lesions, and that the incidence of true high-grade lesions is low among women with a double-negative cotest (cytology and HPV), although likely to be higher in the routine setting compared with study conditions.

Our data identify disadvantages of the current annual Pap smear screening concept in Germany. In participants who attended annually for all five screening visits, the risk of having at least one atypical Pap smear result consistently increased during the second to the fifth screening rounds. If every participant attended all five annual screening rounds (100% attendance), the overall specificity of the Pap smear screening program would be less than 86% for CIN2+. However, only approximately one-third of women attended at least four follow-up visits. The high rate of participants lost during follow-up is only partly explained by migration and women failing to attend routine visits. Another contributing factor is that women frequently changed their gynecologists and were lost to follow-up if the new practice was not participating in the study. As private practices act as independent units, the new practice was usually unaware of previous screening results. Therefore, we cannot exclude the possibility that some cases of high-grade neoplasia may have been missed, but based on the overall follow-up data, the rate of missed diagnosis seems to be very low.

In fact, our study protocol may even underestimate adverse events associated with annual Pap smear tests. We actively discouraged excisional therapy because we assumed a very low risk of CIN2+ in the initially HPV-negative study population and, because we relied completely on voluntary submission of information about any kind of invasive procedures, we cannot exclude a substantial under-reporting of such measures. The practice of histological assessment of women with atypical Pap smears with the direct use of cold knife conization without prior colposcopy is still widespread and adds to the relatively high cost of cervical cancer screening as demonstrated in a recent study in Germany [12]. In addition to cost, several other harmful consequences of overdiagnosis are recognized including pain, inconvenience, anxiety, and procedure-related morbidity [13], including obstetrical adverse effects [14,15].

Guidelines recommend screening intervals can be safely extended to 3 years in women aged over 30 years when HPV and Pap tests are combined [16]. For women aged 30–65 years who want to lengthen the screening interval, the US Preventive Services Task Force (USPSTF) now recommends screening with a combination of cytology and HPV testing every 5 years [17]. Nevertheless, despite national and international guidelines recommending extended screening intervals in women with normal cytology and negative HPV status [18], evidence from the USA has shown that many physicians are reluctant to change from annual screening [16,19]. For example, a nationally representative survey of practice among office-based providers and hospital outpatient departments found that most providers in both settings would continue to perform annual Pap screening for three different clinical scenarios involving women aged 30–60 years with normal Pap and negative HPV test results [16]. Of note, the survey found that only 14% of office-based providers would recommend a Pap screening interval of ≥ 3 years for women aged 30–60 years old, with current Pap and HPV-negative results and two consecutive normal Pap results [16]. Potential reasons suggested for physicians’ resistance to extending Pap screening intervals include the influence of local opinion leaders, concern about less frequent visits for other preventive care examinations, patient preference for annual screening, and loss of financial incentives [19]. Concern has also been raised that HPV co-testing will increase costs unless screening intervals are extended [19]. It is interesting to note from the US survey reported by Saraiya et al. that few physicians recommended another HPV test after 3 years and many thought that not performing any HPV test was an acceptable option [19].

We found a low rate of new HPV infections among women who were initially tested negative for HR-HPV. The rate of new infections was just 3.0% based on HC2 results at 60–68 months of follow-up. This makes repeated HPV-screening with intervals of every 5 years an even more attractive option to prevent cervical cancer. It seems very likely that the HPV-prevalence will be much lower in subsequent screening rounds compared with the prevalence found at first recruitment.

## Conclusion

In conclusion, in women aged over 30 years with normal cytology and a negative HPV test result, Pap smear screening of HPV-negative women more frequently than every 5 years could be potentially harmful and seems to be of little clinical value.

## Competing interests

KUP received speaker’s honorarium from Roche Diagnostics and Becton Dickinson. GB received speaker’s honorarium from Hologic and Roche Diagnostics. TI received institutional grants from Gen-Probe and Hologic. The other authors declare that they have no competing interest.

## Authors’ contributions

TI and KUP were responsible for the overall study design and trial coordination. KUP was responsible for the data analysis, interpretation, writing of the manuscript and data collection. FR, BH, GB were responsible for field work, data collection and interpretation. NB and MB were responsible for statistical analyses and interpretation. AL was responsible for quality control and data collection. All authors read and approved the final manuscript.

## Pre-publication history

The pre-publication history for this paper can be accessed here:

http://www.biomedcentral.com/1471-2407/13/379/prepub
